# Computational Investigation about the Effects of Solvent Polarity and Chalcogen Element Electronegativity on ESIPT Behaviors for the Et_2_N-Substituted Flavonoid

**DOI:** 10.3390/molecules29132957

**Published:** 2024-06-21

**Authors:** Tuo Chang, Fang Yang, Tangyan Chen

**Affiliations:** 1 College of Chinese Medicine, Liaoning University of Traditional Chinese Medicine, Shenyang 110847, China; changtuotcm@163.com; 2 School of Traditional Chinese Medicine, Shenyang Medical College, Shenyang 110034, China; 3 School of Basic Medicine, Shenyang Medical College, Shenyang 110034, China; 19918782825@163.com

**Keywords:** intramolecular hydrogen bond, excited-state intramolecular proton transfer, frontier molecular orbitals, flavone, potential energy surfaces

## Abstract

Inspired by the outstanding nature of flavonoid derivatives in the fields of chemistry and medicine, in this work we mainly focus on exploring the photo-induced properties of the novel Et_2_N-substituted flavonoid (ENF) fluorophore theoretically. Considering the potential photo-induced properties in different solvents and the chalcogen atomic electronegativity-associated photoexcitation, by time-dependent density functional theory (TDDFT) methods we primarily explore the intramolecular hydrogen bonding interactions and photo-induced charge redistribution behaviors. Via comparing geometrical data and the infrared (IR) spectral shifts-associated hydroxy moiety of ENF, we confirm that the intramolecular hydrogen bond O-H···O should be enhanced with facilitating an excited-state intramolecular proton-transfer (ESIPT) reaction. Particularly, the charge reorganization around hydrogen bonding moieties further reveals the tendency of ESIPT behavior. Combined with the construction of the potential energy surface and the search for reaction transition states, we finally confirmed the solvent-polarity-regulated behaviors as well as the chalcogen elements’ electronegativity-dependent ESIPT mechanisms for the ENF fluorophore. We sincerely wish our work could accelerate the further development and applications of flavonoid derivatives.

## 1. Introduction

Natural product flavonoid molecules are widely found in fruits, vegetables and traditional Chinese medicine and are commonly used as food additives [1,2,3]. What is more, because flavonoid molecules have dual fluorescence properties and low toxicity, flavonoid derivatives present a great potential in the design of chemical sensors. Also, flavonoids and flavonols, a class of plant pigments rich in antioxidants, play a pivotal role in imparting vibrant hues of orange, blue, and scarlet to flowers, leaves, and fruits [4,5,6]. Moreover, they exhibit characteristics of fluorescent probes and serve as indicators for metal ions [7]. In medicine, bioflavonoids also have free radical scavenging and antioxidant effects. They also have antithrombotic, protective cardio-cerebrovascular, anti-tumor, anti-inflammatory, and antibacterial effects. To remove alcohol poisoning, they also provide liver protection and other effects. Flavonoids can enhance the non-specific immune function and humoral immune function and so on [8].

It is undeniable that intramolecular hydrogen bond chains in flavonoid derivatives play an important role in photoexcitation dynamics. Especially in probe flavonoid derivatives, the interaction between anions and hydrogen bond donors and acceptors determines the quality of fluorescence detection. As is well known, proton transfer (PT) along with hydrogen bonding is the most fundamental process in the natural world that widely exists in biological and chemical fields [9,10,11]. Considering the effect of photoexcitation, the excited-state intramolecular proton-transfer (ESIPT) process along hydrogen bond chains is particularly important in the design and preparation of optoelectronic devices. The reaction behavior of ESIPT in flavonoid derivatives cannot be ignored. Compared to PT processes in the ground state, ESIPT reactions are generally ultrafast [12], even on the order of several femtoseconds. Generally, the ESIPT belongs to the four-level reaction cycle, which could proceed along with the following sequence: absorption → ESIPT → fluorescence → back to PT in the S_0_ state. Due to the different charge recombination behavior of the reactants and products in ESIPT reactions, the double fluorescence phenomenon and large Stokes shift have also become the signature phenomena of ESIPT reactions [13,14,15]. Just due to the unique luminescent property of ESIPT, many potential applications already involve this reaction, such as molecular switches, fluorescent sensors, LEDs, and so on [16,17,18,19,20].

As mentioned above, because of the double emissions and distinguished biological compatibility, flavonoid derivatives have become significant molecular frames in the fluorescent probe field [21]. The modification and replacement of general chemical groups can change the charge recombination properties of the molecule itself, and then affect the physicochemical properties and luminescence characteristics of the fluorophore itself. Pang and coworkers reported a novel Et_2_N-substituted flavonoid (ENF) fluorophore to explore the effects of substituents on intramolecular charge transfer (ICT) characteristics and fluorescence properties [22]. Particularly, by varying the temperature the emission intensity could be gradually controlled experimentally. As is well known, as a positively charged particle, the proton is capable of transferring in solution, leading to a redistribution of electron density within the studied molecule. This redistribution not only influences interactions with the surrounding environment but also can be controlled by its surroundings. Specifically, when the ESIPT process occurs in condensed dielectric medias, both photochemical and photophysical characteristics could be affected by the dielectric polarization of the surroundings [23]. In addition, in the study of manufacturing organic light-emitting devices, it has been shown that the external quantum efficiency can be improved by oxygen group element doping: that is, due to the heavy atom effect of O, S, and Se atoms, doping oxygen group element atoms can effectively promote the intersystem crossover process between singlet and triplet states, thereby improving the external quantum efficiency and reducing the efficiency roll down. Thus, it is clear that the doping of oxygen group elements has a profound effect on the dynamical processes of molecular excited states.

**Figure 1:**
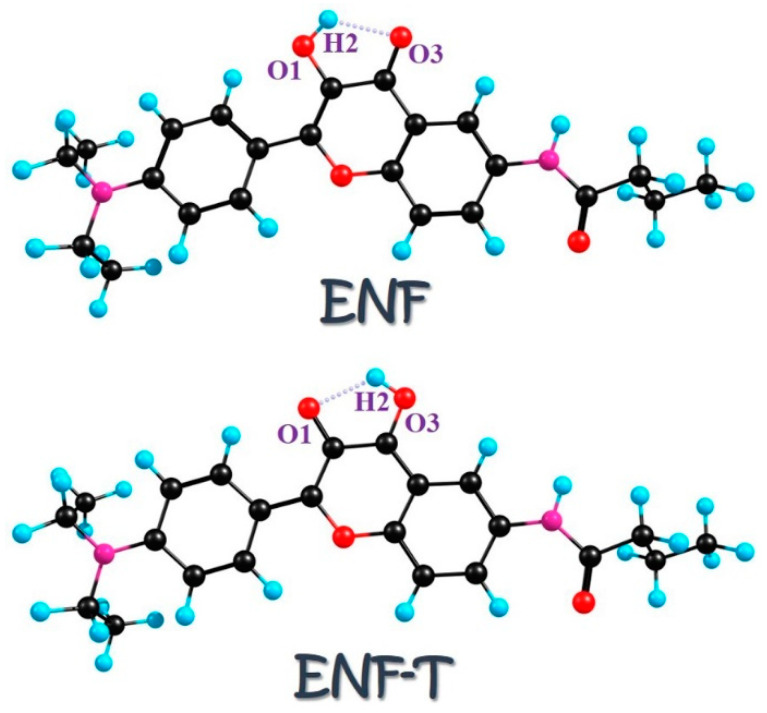
Structures of ENF and the proton-transfer ENF-T tautomer. The intramolecular hydrogen bond is also labeled.

Given the importance of flavonoid derivatives in various fields and the potential regulated manners via surrounding environments or chalcogen elements’ doping, in this work we aim at a theoretical investigation of hydrogen bonding interactions and ESIPT mechanisms for ENF derivatives. The structures of ENF and its proton-transfer ENF-T are shown in Figure 1. The corresponding intramolecular hydrogen bonds O1-H2···O3 of ENF and O1···H2-O3 of ENF-T are marked, respectively. By enhancing the changing behavior of excited hydrogen bonds in different solvents after photoexcitation and the barrier size of chalcogen elements’ doping-dependent ESIPT, we present the solvent-polarity-dependent and atomic electronegativity-regulated ESIPT mechanism for the ENF system.

## 2. Results and Discussions

### 2.1. Solvent Polarity-Associated Excited-State Behaviors

The elegant depiction of ENF and its proton-transfer tautomer ENF-T can be observed in Figure 1. In order to investigate the potential molecular behaviors in excited states resulting from solvent effects (i.e., solvent polarity), all simulations related to the ENF system were conducted in three solvents (cyclohexane, dichloromethane, and acetonitrile). In Table S1, we list the relative energies of ENF and ENF-T forms in three solvents in both S_0_ and S_1_ states. Clearly, the S_0_-state ENF and ENF-T cannot coexist due to the lower energies of optimized ENF forms in three solvents. Thus, all the simulations in this work have been carried out based on the ENF configurations in all solvents. It is imperative to probe into the infrared (IR) vibrational spectra of all the related molecular structures in solvents prior to delving into the specific kinetics of excited-state reactions, as the geometric stability of the configurations mentioned in this study can be ensured by non-imaginary frequency results obtained from the IR vibrational spectra across all excited states. Herein, our primary focus lies on scrutinizing variations in IR vibrational spectral behaviors associated with the intramolecular hydrogen bond O1-H2···O3 of the ENF fluorophore, given that proton-transfer reactions can only occur alongside pre-existing hydrogen bond networks [24,25,26].

It is well known that the charge recombination behavior caused by photoexcitation can reflect the trend of the excited-state reaction of molecules to a large extent. Therefore, we firstly use frontier molecular orbitals (MOs) to examine the case of photo-induced charge reorganization. Based on the optimized S_0_-state structure, we mainly calculate the excitation behavior of the first six excited states for the ENF fluorophore. Since highly excited states correspond to insignificant oscillator strengths, we only list the calculated results of the first three vertical excitation behaviors (S_0_ → S_1_, S_0_ → S_2_, and S_0_ → S_3_) in Table 1. In dichloromethane, the maximum absorption peak we calculated is 433.02 nm, which is consistent with the experimental report (~420 nm) [22]. This also preliminarily confirms the rationality of our calculation method. In the three different solvents, we can find that with the increase in the polarity of the solvent, the maximum absorption peak position presents a small redshift, which reflects that the polarity of the solvent has a certain effect on its photoexcitation behavior.

**Figure 2:**
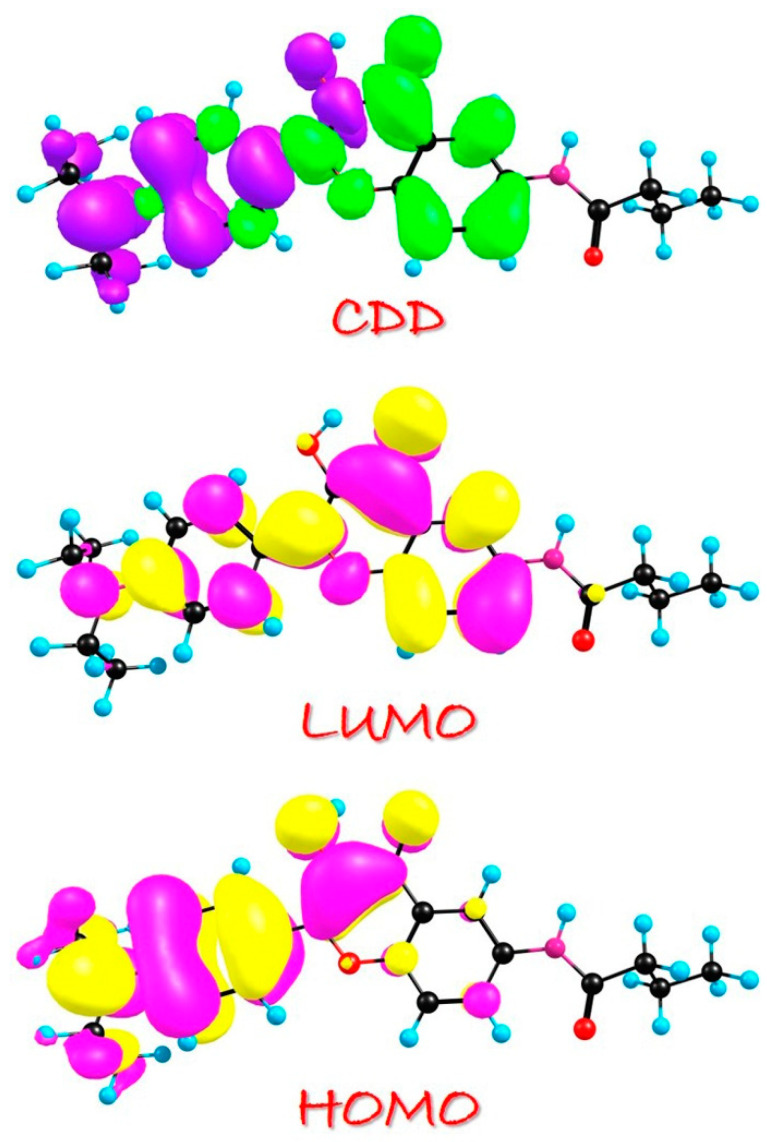
HOMO and LUMO orbitals for the ENF fluorophore. The CDD result between S_1_ and S_0_ of ENF is also provided.

To gain a deeper insight into the redistribution of charge and electrons, we have also included the visualization of the molecular orbitals (MOs) of ENF in dichloromethane solvent in Figure 2. Herein, we want to mention that the molecular orbitals (MOs) of ENF in the three solvents are almost the same; thus, only the corresponding MOs’ results in dichloromethane solvent are displayed. It is worth emphasizing that the S_0_ → S_1_ transition observed in all three solvents for the ENF system predominantly arises from the HOMO-LUMO transition, as evidenced by CI (%) values exceeding 98% in Table 1. Consequently, only these two orbitals of ENF are depicted in Figure 2. Evidently, the S_0_ → S_1_ behavior corresponds to the ππ*-type transition. During the HOMO → LUMO transition, the most intriguing aspect lies in the charge-altering phenomenon across O1-H2···O3 moieties. Our primary focus revolves around elucidating the charge reorganization encompassing both the hydrogen bonding donor and acceptor regions where the intramolecular charge transfer (ICT) occurs clearly. Additionally, in analyzing the results of charge density difference (CDD), the green color denotes an augmented distribution of electron densities, while violet indicates a diminished distribution of electron densities. In fact, the charge density difference (CDD) results between the excited state and ground state could be easily evaluated as the following formula:

$$∆\rho \left(r\right)=\rho^{elec}\left(r\right)-\rho^{hole}(r)$$

The observed increase/decrease in the electron density should be the S_0_-to-S_1_ absorption process. Clearly, a discernible shift in electron densities involved in the hydrogen bonding moieties of ENF from O1 towards O3 moieties is observed upon photoexcitation. Moreover, the phenomenon of ESIPT leads to a substantial alteration in the distribution of electronic charge density on heavy atoms induced by photoexcitation.

**Figure 3:**
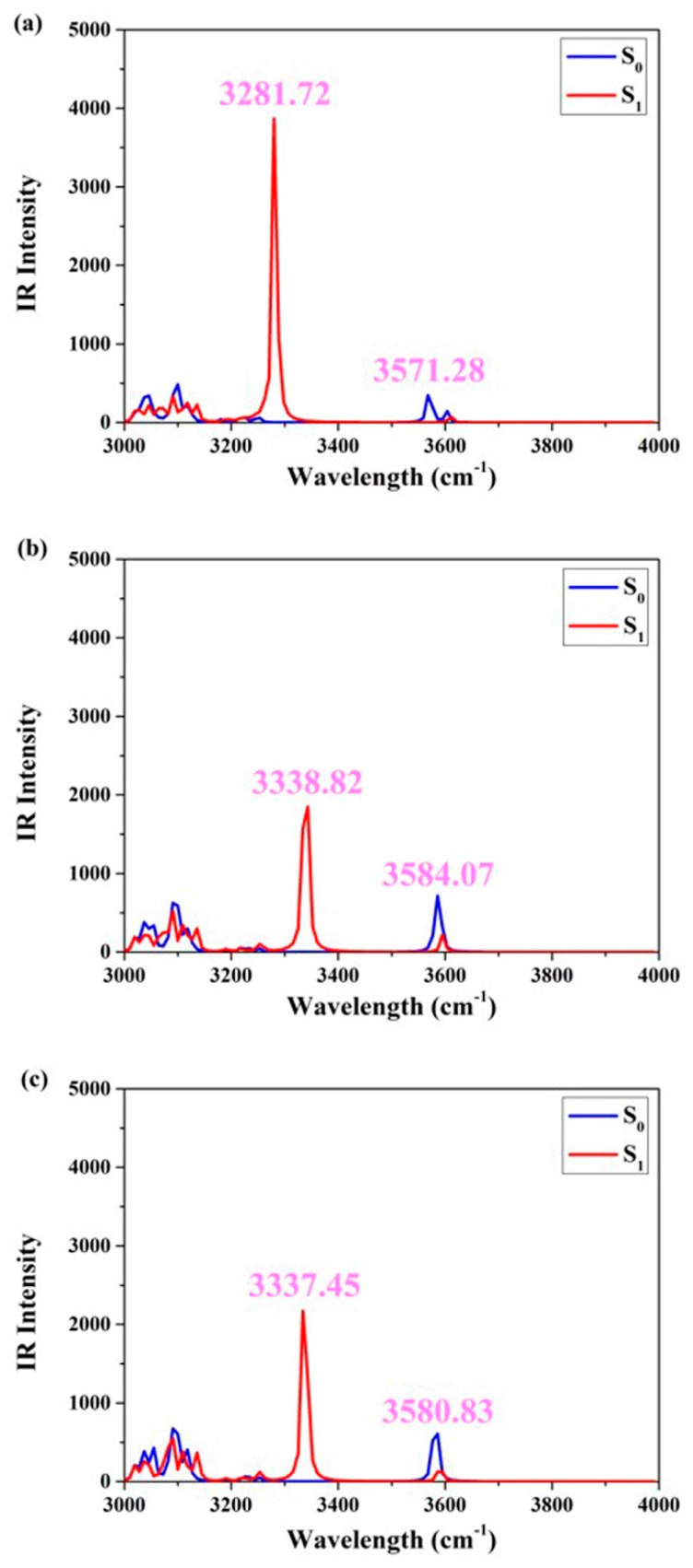
Simulated S_0_-state and S_1_-state IR associated with the O-H stretching vibrational mode for ENF in (**a**) cyclohexane, (**b**) dichloromethane, and (**c**) acetonitrile solvents, respectively.

In order to facilitate a more comprehensive comparison of the similarities and disparities between hydrogen bonds in the S_0_ and S_1_ states, Figure 3 displays the infrared vibrational spectral peaks corresponding to the O1-H2 stretching vibration in the three different solvents. Evidently, within cyclohexane, dichloromethane, and acetonitrile solvents, the infrared peaks associated with the elongation vibration of O1-H2 in the S_0_ state are measured at 3571.28 cm^−1^, 3584.07 cm^−1^, and 3580.83 cm^−1^, respectively. Subsequent to photo-induced excitation, these same O1-H2 stretching vibrations exhibit an altered infrared peak position in the S_1_ state: specifically, at 3281.72 cm^−1^, 3338.82 cm^−1^, and 3337.45 cm^−1^, respectively. The O1-H2 stretching vibration in the three solvents exhibits a conspicuous redshift of the IR peak, indicating that the S_1_ state is highly favorable for enhancing the intramolecular hydrogen bond interaction [24,25,26]. To be more precise, this redshift measures 289.56 cm^−1^ (cyclohexane), 245.25 cm^−1^ (dichloromethane), and 243.38 cm^−1^ (acetonitrile), respectively. Furthermore, it reflects the alteration in solvent polarity and highlights the distinct influence of photoexcitation on hydrogen bonding. Notably, the most prominent redshift observed in nonpolar aprotic solvents underscores their significant role in promoting excited-state reactions for the ENF fluorophore.

Furthermore, as presented in Table 2, we showcase the elementary structural parameters of optimized ENF structures in solvents, encompassing bond distances and bond angles. Additionally, the relative geometrical outcomes of the proton-transfer tautomer (ENF-T) are outlined in Table S2. Upon photoexcitation, when compared to the S_0_ state mentioned in Table 1, it becomes apparent that in the S_1_ state there is an elongation observed in the bond length of hydroxyl O1-H2 while simultaneously a reduction can be witnessed in hydrogen bond distance (H2···O3), ultimately leading to an enlargement of the bond angle Δ(O1-H2···O3). Specifically, the distance of O1-H2 increased by 0.0171 Å (cyclohexane), 0.0139 Å (dichloromethane), and 0.0137 Å (acetonitrile), respectively, while the distance of H2···O3 decreased by 0.1721 Å, 0.1635 Å, and 0.1597 Å, respectively. At the same time, the bond angles increased by 6.22° (cyclohexane), 5.99° (dichloromethane), and 5.86° (acetonitrile), respectively. The occurrence of such structural changes further suggests that the intramolecular hydrogen bonding interaction can be enhanced through photoexcitation [24,25,26].

To further elucidate and compare the extent of strengthening in excited-state hydrogen bonding across different solvents, we also direct our attention towards investigating the core-valence bifurcation (CVB) index based on the electron localization function (ELF) [27]. The exquisite revelation of the hydrogen bonding interaction can be achieved by exploring the parameters ELF(C-V,D) and ELF(DH-A) using Multiwfn [28]. By employing the formula proposed by Fuster and colleagues (i.e., CVB index = ELF(C-V,D) − ELF(DH-A)) [27], the commendable comparison of hydrogen bond strength under different solvent conditions can be accomplished through the utilization of the CVB index. As a fundamental principle, it is well known that a more negative CVB index signifies a stronger hydrogen bond interaction [27]. The CVB index of the S_1_ state, as presented in Table 3, is conspicuously more negative than that of the S_0_ state, thereby corroborating the aforementioned conclusion regarding hydrogen bond reinforcement in excited states. Furthermore, it becomes evident that a less polar solvent yields a more negative CVB index, signifying that a nonpolar solvent environment fosters an even greater enhancement of hydrogen bonding in the S_1_ state. Consequently, we can tentatively anticipate that the dynamic processes occurring in the excited state would be better facilitated within nonpolar solvents for the ENF compound.

Additionally, we employed the atom-in-molecule approach to scrutinize the electron density distribution of the ENF compound across the three solvents. The bond critical point (BCP) parameters linking acceptor and hydrogen atoms are presented in Table S3. Evidently, robust hydrogen bonding interactions exist between S_0_ and S_1_ of the ENF compound within all three solvents. As is well known, the electron density (ρ(r)) plays a pivotal role in determining the strength of chemical bonds. It has come to our attention that the ρ(r) values in the S_1_ state can be more negative compared to those in the S_0_ state, thereby demonstrating an intensified hydrogen bonding effect upon photoexcitation. Furthermore, we place equal emphasis on both ρ(r) and hydrogen bonding energy (E_HB_) for ENF analysis. The predicted E_HB_ can be calculated using E_HB_ ≈ −223.08 × ρ(r) + 0.7423 [29]. Clearly, the higher values of both ρ(r) and E_HB_ observed in cyclohexane solvent suggest a heightened strength of hydrogen bonding in nonpolar solvents, which effectively enhances the ESIPT reactions of the ENF fluorophore.

**Figure 4:**
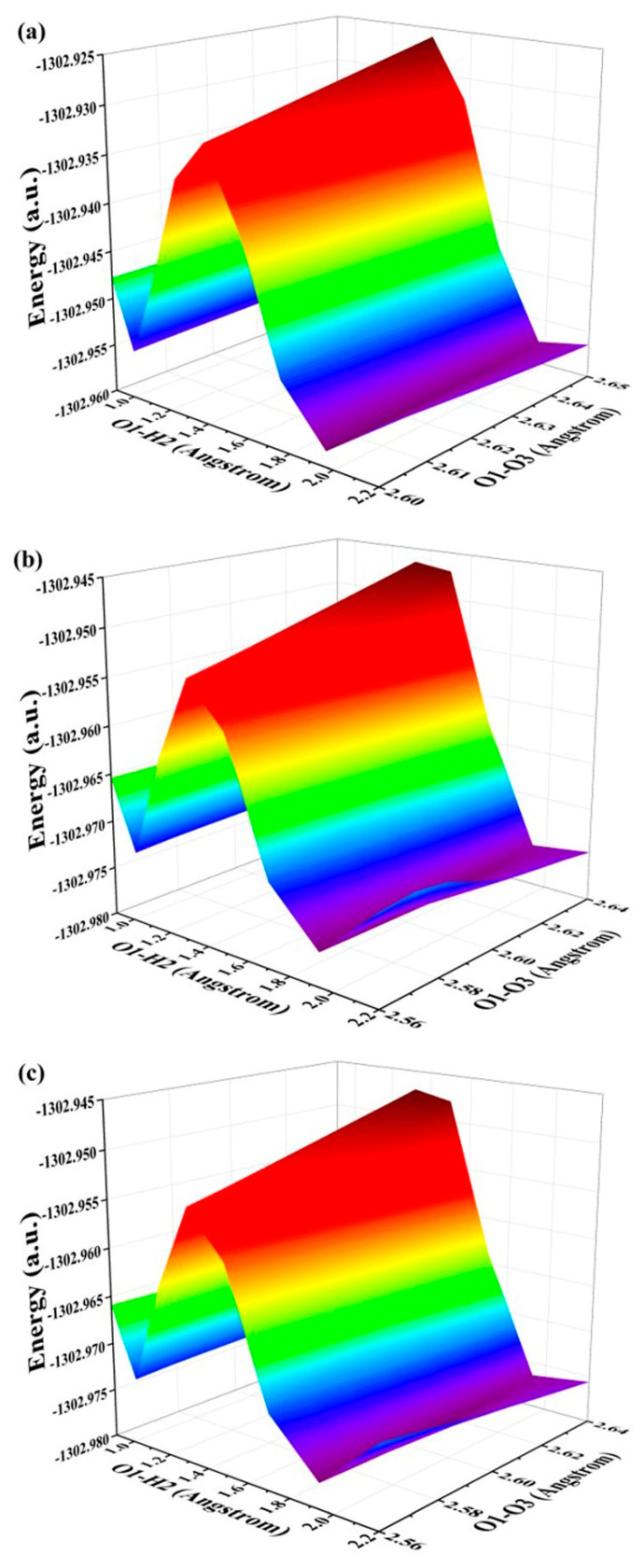
Constructed S_1_-state PESs along with both O1-H2 and O1-O3 bond lengths for the ENF fluorophore in (**a**) cyclohexane, (**b**) dichloromethane, and (**c**) acetonitrile solvents, respectively.

**Figure 5:**
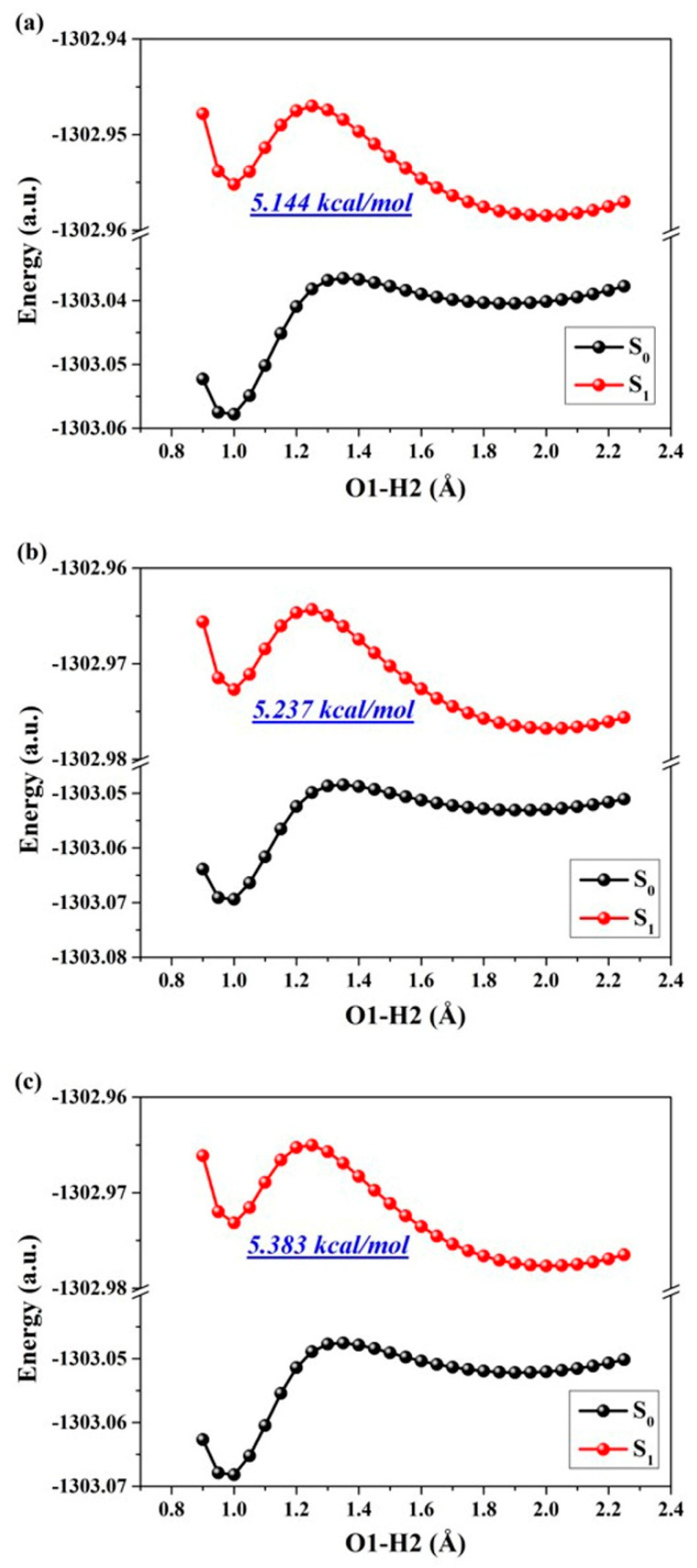
The constructed S_0_-state and S_1_-state PECs for ENF in (**a**) cyclohexane, (**b**) dichloromethane, and (**c**) acetonitrile solvents, respectively.

The aforementioned analysis and discussion have unequivocally demonstrated that the reinforcement of excited hydrogen bonding and recombination of photo-induced charges can effectively unveil the ESIPT behavior for ENF fluorophore. Consequently, in this section our focus lies on exploring and elucidating the specific mechanisms governing the excited state. To quantitatively depict the reaction processes and barriers in these states, we investigate the behaviors of ESIPT reactions by constructing potential energy surfaces (PESs). It is well recognized that PESs pose challenges to normal chemical reactions in excited states due to one or more geometric changes [13,18,30,31,32,33]. Regarding the hydrogen bond, the O1-H2···O3 interaction can be classified as a five-membered ring type. Empirically, this specific type of hydrogen bond often undergoes photoexcitation-induced changes in both the proton donor-recipient distance and the initial form-proton-transfer tautomer distance. To comprehensively investigate ESIPT behaviors, we employed a restrictive optimization method to construct S_1_-state PESs around the hydrogen bonding region using two coordinates (specifically by maintaining fixed values for O1-H2 and O1-O3 distances) (as depicted in Figure 4). The step size for varying O1-O3 is set at 0.01 Å, while that for adjusting O1-H2 is set at 0.05 Å. The constructed PESs comprehensively encompass the optimized S_1_-state of ENF and its proton-transfer tautomer (ENF-T) in the three solvents. Qualitatively speaking, the overall energy along the O1-O3 coordinate direction remains relatively stable for these structures. Therefore, it is reasonable to focus solely on the changes in energy along the O1-H2 coordinate during the ESIPT process of the ENF fluorophore. Thus, we further separately provide the potential energy curves (PECs) of the ESIPT reaction of ENF in the three solvents along with the O1-H2 bond distance (seen in Figure 5). As labeled in Figure 5, the values of the potential energy barriers reveal that along with the decrease in solvent polarity the ESIPT reaction becomes more and more effortless. Consistent with the above analysis, hydrogen bond interaction and the ESIPT reaction mechanism regulated by solvent polarity can be obtained.

### 2.2. Chalcogen Atomic Electronegativity-Regulated Excited-State Processes

**Figure 6:**
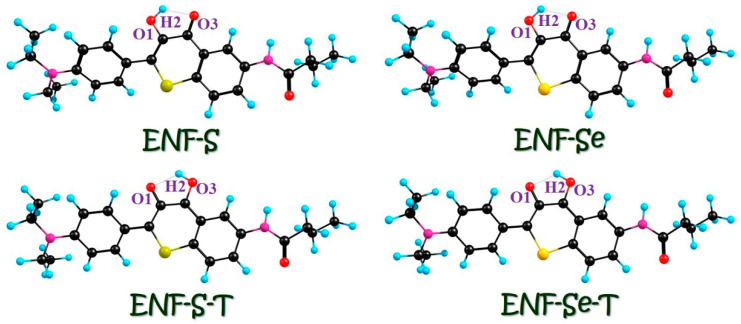
Structures of ENF-S and ENF-Se as well as the proton-transfer ENF-S-T and ENF-Se-T tautomers. Intramolecular hydrogen bonds are also labeled, respectively. Black: C atoms; Blue: H atoms; Red: O atoms; Violet: N atoms; Yellow: Se; Claybank: S.

In this section, we mainly focus on the effect of atomic electronegativity on hydrogen bond strength and ESIPT behaviors. Based on DFT and TDDFT methods, we optimize the ENF-S and ENF-Se fluorophores after S/Se substitution (seen in Figure 6) in dichloromethane solvent. Correspondingly, the proton-transfer ENF-S-T and ENF-Se-T forms are also shown. Herein, we also present the relative energies of ENF-S and ENF-Se as well as their proton-transfer tautomers in Table S4. Also, the S_0_-state ENF-S and ENF-Se as well as the proton-transfer ENF-S-T and ENF-Se-T cannot coexist due to the lower energies in dichloromethane solvent. Similarly, we firstly perform the IR spectral simulations for the optimized ENF-S and ENF-Se compounds in both S_0_ and S_1_ states (displayed in Figure S1). The infrared peaks associated with the stretching vibration of O1-H2 in the S_0_ state are measured at 3540.18 cm^−1^ and 3522.62 cm^−1^ for ENF-S and ENF-Se, respectively. Following the photoexcitation, these same O1-H2 stretching vibrations exhibit the obvious infrared peak position in the S_1_ state: 3259.19 cm^−1^ and 3237.47 cm^−1^, respectively. Obviously, the distinct redshift from S_0_ to S_1_ demonstrates the strengthening hydrogen bonding interaction in the S_1_ state [24,25,26]. Comparing with NEF from O to S to Se, we could find that the value of redshift is, respectively, 245.25 cm^−1^, 280.99 cm^−1^, and 285.55 cm^−1^. This result indicates that along with the decrease in atomic electronegativity (O → S → Se) the S_1_-state hydrogen bond is strengthened even more strongly.

Moreover, we also list the optimized geometrical parameter of ENF-S and ENF-Se in S_0_ and S_1_ states in Table 4. Analogously, by comparison, it is not difficult to find that the distance of the hydroxyl O1-H2 group should be lengthened under photoexcitation, while the distance of the hydrogen bond H2···O3 could be shortened. At the same time, the bond angle also becomes larger in the S_1_ state. Consistent with the IR results of redshift, these results indicate that the photoexcitation causes the strengthening of the hydrogen bond in the S_1_ state [24,25,26]. In order to investigate the strength of hydrogen bond interactions with different atomic electronegativities (O → S → Se), herein we still perform the simulations of the CVB index for ENF-S and ENF-Se fluorophores. As listed in Table S5, the ELF(C-V, D), ELF(DH-A), and CVB are provided. In fact, it is interesting to find that the S_0_-state CVB values of ENF, ENF-S, and ENF-Se decrease (0.0229 → 0.0015 → −0.0084). It indicates that as the atomic electronegativity changes (O → S → Se), the S_0_-state hydrogen bond also becomes stronger. For the case of the S_1_ state, the CVB reduces from −0.0167 (O) to −0.0455 (S) to −0.0539 (Se), which clearly demonstrates that S_1_-state hydrogen bonding interactions should be enhanced along with the decrease in chalcogen atomic electronegativity [27]. To provide the quantitative hydrogen bonding energies, we also pay attention to the BCP results for ENF-S and ENF-Se compounds. Along with hydrogen bond O1-H2···O3, S_0_-state and S_1_-state ρ(r) and the predicted E_HB_ are listed in Table 5. Mainly focusing on the E_HB_ values of ∆E (S_1_-S_0_), compared with Table S3, the ∆E (S_1_-S_0_) is −2.2553, −2.3639, and −2.5052 kcal/mol for ENF, ENF-S, and ENF-Se, respectively. Apparently, the lower the electronegativity of oxygen group elements, the greater the change in hydrogen bonding energy.

**Figure 7:**
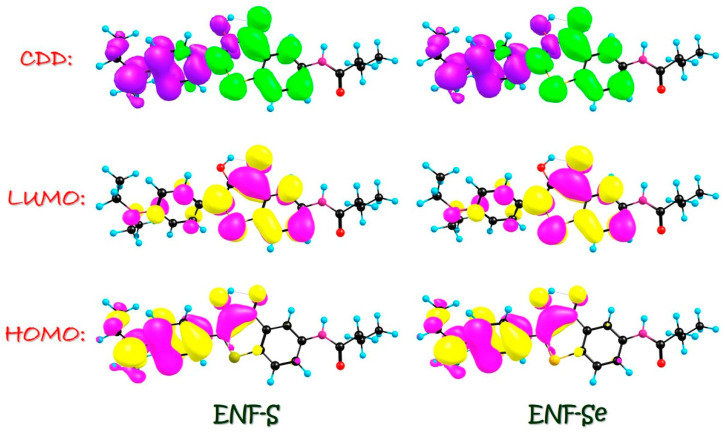
HOMO and LUMO orbitals for ENF-S and ENF-Se fluorophores in dichloromethane solvent. The CDD results between S_1_ and S_0_ of ENF-S and ENF-Se are also provided in dichloromethane solvent. Violet in CDD stands for hole, while the green in CDD means electron. Deep pink and yellow in HOMO & LUMO mean the distributions of electronic clouds.

Charge reorganization, as the primary driving force, is of utmost importance in determining the behaviors and properties of excited states. It plays a pivotal role in various scientific fields such as chemistry, physics, and materials science. Furthermore, studying charge reorganization is crucial for developing new strategies to enhance energy storage technologies. Understanding charge reorganization is essential for unraveling biological processes involving excited states. For instance, it plays a vital role in photosynthesis by facilitating the efficient energy transfer between pigment molecules during light absorption. Therefore, this study focuses on investigating the photo-induced absorption aspects of ENF, ENF-S, and ENF-Se fluorophores. The vertical excitation results of ENF-S and ENF-Se in dichloromethane solvent are provided in Table 6. Combined with Table 1, it could be found that the absorption peak of ENF, ENF-S, and ENF-Se is, respectively, 433.02, 457.07, and 465.80 nm in dichloromethane solvent. It indicates that the steady-state absorption spectra could be also affected by chalcogen elements’ substitutions: that is, absorption peaks occur redshift with the decrease in atomic electronegativity. Also, in Table 6 the S_0_ → S_1_ transition of ENF-S and ENF-Se principally corresponds to HOMO-LUMO, with orbital transition contributions more than 97%. Similar with the ENF fluorophore, the ππ*-type transition could be also found during the HOMO → LUMO transition (seen in Figure 7). Also, in the analysis of CDD maps it could be found that electron densities shift from O1 to O3 moieties upon photoexcitation. 

**Figure 8:**
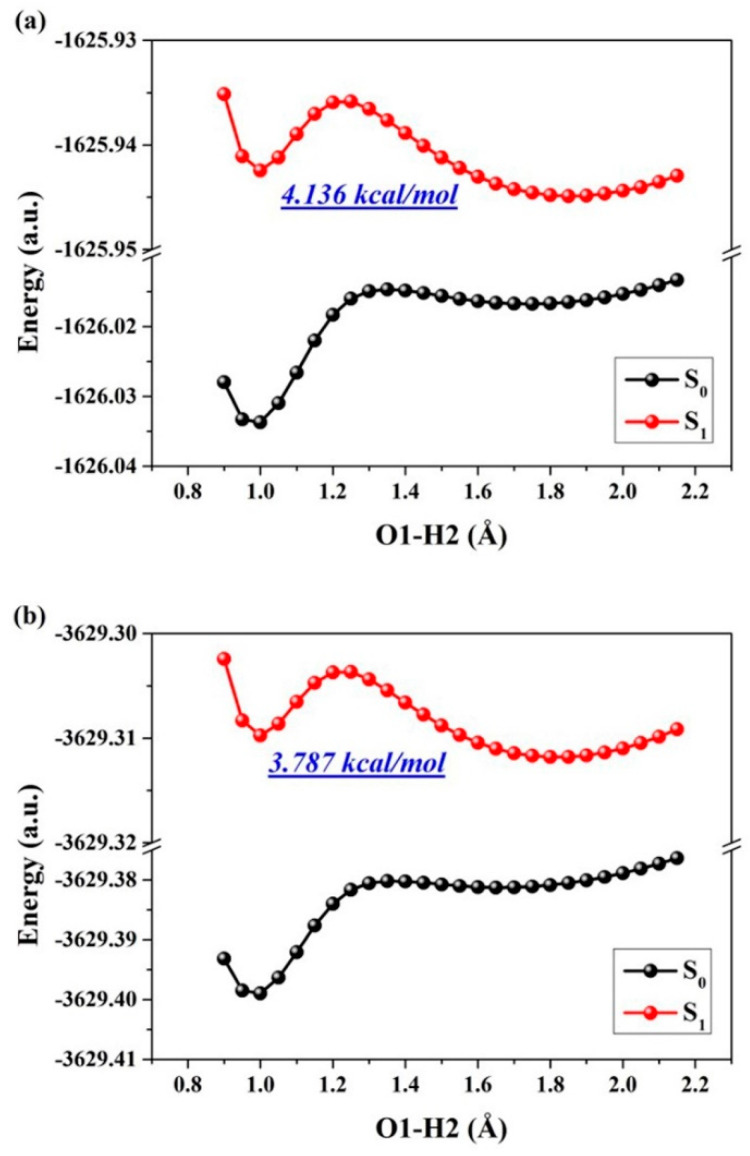
The constructed S_0_-state and S_1_-state PECs for (**a**) ENF-S and (**b**) ENF-Se in dichloromethane solvent, respectively.

The determination of reaction processes and barriers in excited states can be achieved quantitatively by constructing PECs through a restrictive optimization approach, thereby facilitating the investigation of ESIPT reaction behaviors. It is widely acknowledged that PECs present challenges to conventional chemical reactions in excited states due to one or more alterations in geometry [13,18,30,31,32,33]. By employing a rigorous optimization method, we successfully constructed PECs while preserving an elongated O1-H2 bond distance ranging from 0.90 Å to 2.20 Å in increments of 0.05 Å, encompassing all photo-induced configurations (as illustrated in Figure 8). From a qualitative perspective, it becomes apparent that higher potential barriers hinder the PT reaction in S_0_ state, whereas lower barriers in S_1_ suggest the facile occurrence of ESIPT. Importantly, the thermodynamic feasibility of ESIPT reactions for derivatives of ENF, ENF-S, and ENF-Se is supported by their respective potential energy barriers: namely, 5.237 kcal/mol, 4.136 kcal/mol, and 3.787 kcal/mol. Therefore, considering kinetic aspects, we can assert that low atomic electronegativity promotes ESIPT reaction for these ENF derivatives. We speculate that the six-membered ring belonging to O, S, and Se will squeeze the distance between O1 and O3 relative to the atomic radius of O to S to Se increasing gradually. For this reason, the chalcogen atomic electronegativity-associated ESIPT mechanism could be revealed.

## 3. Theoretical Calculation Methods

In this work, the Gaussian 16, Revision C. 01, software package was utilized to perform all theoretical simulations [34]. The B3LYP functional and TZVP basis set were employed, taking into account the D3 version of Grimme’s dispersion for the better consideration of weak interactions [35,36], based on which the relative properties of S_0_ and S_1_ states for ENT derivative fluorophores could be determined. To ensure that the obtained structure is a local minimum, its frequency was calculated. Herein, for simulating the infrared (IR) spectra, the scale factor adopted in this work is 0.999, which is the closest approximation to the B3LYP/TZVP theoretical level. In addition, we selected cyclohexane, dichloromethane, and acetonitrile as solvents based on the solvation model of density (SMD) method [37]. To thoroughly investigate the reaction mechanism of ESIPT, we calculated and analyzed the infrared vibrational behaviors, core-valence bifurcation (CVB) indexes, bond critical point (BCP) parameters, frontier molecular orbitals (MOs), and potential energy surfaces (PESs) according to optimized configurations. For further checking the reaction kinetics, by Berny optimization method [38] the transition state forms are also searched that own only one imaginary frequency. 

## 4. Conclusions

In conclusion, our research primarily focuses on exploring the potential impacts of solvent polarity and atomic electronegativity on photo-induced hydrogen bonding interactions (specifically denoted as O1-H2···O3) as well as investigating the behavior of ESIPT in relation to the ENF fluorophore. To thoroughly investigate these influences from their surrounding environments, we have carefully chosen three aprotic solvents with diverse polarities. Furthermore, to delve into the effects caused by atomic electronegativity variations, we have meticulously designed and optimized derivatives including ENF, ENF-S, and ENF-Se fluorophores. By comparing the optimized structural parameters and examining the IR spectral values associated with the O1-H2 stretching mode, we have preliminarily determined the hydrogen bonding interaction of ENF in various solvents. Furthermore, upon photoexcitation the hydrogen bond strengthening effect is observed in the derivatives (ENF, ENF-S, and ENF-Se). Additionally, through an exploration of the CVB index related to hydrogen bonding in four solvents, we can confidently conclude that the enhancement of hydrogen bonds will be more pronounced in nonpolar aprotic solvents. By investigating excitation, HOMO-LUMO orbitals, and the CDD map, we have observed the promotion of intramolecular charge transfer (ICT) and the subsequent redistribution of charges that significantly enhances the excited-state intramolecular proton-transfer (ESIPT) tendency. Moreover, through constructing potential energy curves (PECs) with constrained optimization, we offer comprehensive insights into how solvent polarity controls and atomic electronegativity influences ESIPT dynamics. This study not only elucidates the photo-induced hydrogen bonding behaviors but also proposes a regulatory approach to modulate the ESIPT behavior of 2P3HBQ and its derivatives.

## Figures and Tables

**Table 1:** Simulated photo-induced excitation data for ENF in cyclohexane, dichloromethane, and acetonitrile solvents via the TDDFT method.

	Transition	*λ*	*f*	Composition	CI (%)
cyclohexane	S_0_ → S_1_	424.85	0.8608	H → L	98.90
	S_0_ → S_2_	341.99	0.0838	H-1 → L	92.54
	S_0_ → S_3_	310.26	0.0751	H → L + 1	85.98
dichloromethane	S_0_ → S_1_	433.02	0.8661	H → L	99.03
	S_0_ → S_2_	345.36	0.0814	H-1 → L	92.90
	S_0_ → S_3_	312.81	0.1226	H → L + 1	88.45
acetonitrile	S_0_ → S_1_	435.80	0.8547	H → L	98.97
	S_0_ → S_2_	346.24	0.0749	H-1 → L	92.75
	S_0_ → S_3_	313.22	0.1333	H → L + 1	87.73

**Table 2:** Computational structural results (bond lengths (Å) and bond angles Δ(O1-H2···O3) (°)) associated with hydrogen bond O1-H2···O3 of ENF in cyclohexane, dichloromethane, and acetonitrile solvents in both S_0_ and S_1_ states.

	Cyclohexane		Dichloromethane		Acetonitrile	
	S_0_	S_1_	S_0_	S_1_	S_0_	S_1_
O1-H2	0.9779	0.9950	0.9775	0.9914	0.9774	0.9911
H2···O3	2.0143	1.8422	2.0472	1.8837	2.0428	1.8831
Δ	118.98	125.20	117.67	123.66	117.85	123.71

**Table 3:** Simulated ELF(C-V,D), ELF(DH-A), and CVB parameters related to O1-H2···O3 of ENF in cyclohexane, dichloromethane, and acetonitrile in S_0_ and S_1_ states.

	Cyclohexane		Dichloromethane		Acetonitrile	
	S_0_	S_1_	S_0_	S_1_	S_0_	S_1_
ELF(C-V,D)	0.0950	0.0971	0.0948	0.0967	0.0948	0.0963
ELF(DH-A)	0.0774	0.1269	0.0719	0.1134	0.0709	0.1122
CVB index	0.0176	−0.0298	0.0229	−0.0167	0.0239	−0.0159

**Table 4:** Computational structural results (bond lengths (Å) and bond angles Δ(O1-H2···O3) (°)) associated with hydrogen bond O1-H2···O3 of ENF-S, ENF-S-T, ENF-Se, and ENF-Se-T in dichloromethane solvent in both S_0_ and S_1_ states.

	ENF-S		ENF-S-T		ENF-Se		ENF-Se-T	
	S_0_	S_1_	S_0_	S_1_	S_0_	S_1_	S_0_	S_1_
O1-H2	0.9796	0.9955	1.7341	1.8603	0.9802	0.9963	1.6615	1.8196
H2-O3	1.9319	1.7855	1.0057	0.9876	1.8907	1.7606	1.0152	0.9893
Δ	120.10	126.56	126.74	122.01	121.09	127.26	129.08	123.15

**Table 5:** The electron density (ρ) based on the BCP parameter and predicted bonding energy (E_HB_) in ENF-S and ENF-Se fluorophores in S_0_ and S_1_ states in dichloromethane solvent.

Solvents	S_0_		S_1_		∆ρ (S_1_-S_0_)	∆E (S_1_-S_0_)
	ρ	E_HB_	ρ	E_HB_	ρ	E_HB_
ENF-S	0.03157	−6.3003	0.04217	−8.6642	0.01060	−2.3639
ENF-Se	0.03453	−6.9584	0.04575	−9.4636	0.01120	−2.5052

**Table 6:** Simulated photo-induced excitation data for ENF-S and ENF-Se in dichloromethane solvent by the TDDFT method.

	Transition	*λ*	*f*	Composition	CI (%)
ENF-S	S_0_ → S_1_	457.07	0.5598	H → L	98.64
	S_0_ → S_2_	376.54	0.0805	H-1 → L	94.77
	S_0_ → S_3_	325.07	0.0712	H → L + 1	91.89
ENF-Se	S_0_ → S_1_	465.80	0.5307	H → L	97.91
	S_0_ → S_2_	395.71	0.0964	H-1 → L	95.40
	S_0_ → S_3_	324.00	0.0454	H → L + 1	65.51

## Data Availability

Data are available on request from the corresponding author.

## Supplementary Materials

The following supporting information can be downloaded at: https://www.mdpi.com/article/10.3390/molecules29132957/s1. Table S1: The optimized energy (a.u.) for ENF and ENF-T forms in cyclohexane, dichloromethane, and acetonitrile solvents in both S_0_ and S_1_ states. Table S2: Computational structural results (bond lengths (Å) and bond angles Δ(O1···H2-O3) (°)) associated with hydrogen bond O1···H2-O3 of ENF-T in cyclohexane, dichloromethane, and acetonitrile solvents in both S_0_ and S_1_ states. Table S3: The electron density (ρ) based on the BCP parameter and predicted bonding energy (E_HB_) in the ENF fluorophore in S_0_ and S_1_ states in cyclohexane, dichloromethane, and acetonitrile solvents. Table S4: Simulated ELF(C-V,D), ELF(DH-A), and CVB parameters related to O1-H2···O3 of ENF-S and ENF-Se in dichloromethane solvent in S_0_ and S_1_ states. **Table S5**. Simulated ELF(C-V,D), ELF(DH-A), and CVB parameters related to O1-H2···O3 of ENF-S and ENF-Se in dichloromethane solvent in S_0_ and S_1_ states. Figure S1: Simulated S_0_-state and S_1_-state IR associated with the O-H stretching vibrational mode for (a) ENF-S and (b) ENF-Se in dichloromethane solvent, respectively. The coordinates of all the transition state forms.
