# Supplementary material for: Computational Investigation about the Effects of Solvent Polarity and Chalcogen Element Electronegativity on ESIPT Behaviors for the Et2N-Substituted Flavonoid
Source: Molecules. 2024 Jun 21;29(13):2957. doi: 10.3390/molecules29132957 (PMC11243531; doi:10.3390/molecules29132957)
Supplement: Supplementary file 1 [file molecules-29-02957-s001.zip › molecules-3036277-supplementary.pdf]

Table S1. The relative energies (kcal/mol) for ENF and ENF-T forms in three solvents in both S_0 and S_1 state. Herein, we select the lowest S_0-state ENF as the energy zero in cyclohexane, dichloromethane and acetonitrile solvents, respectively.

	cyclohexane	dichloromethane	acetonitrile
ENF (S_0)	0.000	0.000	0.000
ENF-T (S_0)	11.114	10.440	10.282
ENF (S_1)	64.608	60.880	59.834
ENF-T (S_1)	62.572	58.335	57.038

Table S2. Computational structural results (bond lengths (Å) and bond angles Δ(O1···H2-O3) (°)) associated with hydrogen bond O1···H2-O3 of ENF-T in cyclohexane, dichloromethane and acetonitrile solvents in both S_0 and S_1 states.

	cyclohexane		dichloromethane		acetonitrile	
	S_0	S_1	S_0	S_1	S_0	S_1
O1···H2	1.8754	1.9912	1.9144	2.0115	1.9122	2.0124
H2-O3	0.9949	0.9828	0.9918	0.9824	0.9917	0.9823
Δ	123.00	118.76	121.59	117.99	121.73	118.02

Table S3. The electron density (ϱ) based on BCP parameter and predicted bonding energy (E_{HB}) in ENF fluorophore in S_0 and S_1 states in cyclohexane, dichloromethane and acetonitrile solvents.

Solvents	S_0		S_1		Δϱ (S_1- S_0)	ΔE (S_1- S_0)
	ϱ	E_{HB}	ϱ	E_{HB}	ϱ	E_{HB}
cyclohexane	0.02643	-5.1537	0.03811	-7.7593	0.01168	-2.6056
dichloromethane	0.02474	-4.7767	0.03485	-7.0320	0.01011	-2.2553
acetonitrile	0.02468	-4.7633	0.03419	-6.8848	0.00951	-2.1215

Table S4. The relative energies (kcal/mol) for ENF-S and ENF-Se forms in dichloromethane solvent in both S_0 and S_1 state. Herein, we select the lowest S_0-state ENF-S and ENF-Se as the energy zero, respectively.

	ENF-S (S_0)	ENF-S-T (S_0)	ENF-S (S_1)	ENF-S-T (S_1)
dichloromethane	0.000	10.841	57.459	55.905
	ENF-Se (S_0)	ENF-Se-T (S_0)	ENF-Se (S_1)	ENF-Se-T (S_1)
dichloromethane	0.000	11.286	56.174	54.871

Table S5. Simulated ELF(C-V,D), ELF(DH-A), and CVB parameters related to O1-H2···O3 of ENF-S and ENF-Se in dichloromethane solvent in S_0 and S_1 states.

	ENF-S		ENF-Se	
	S_0	S_1	S_0	S_1
ELF(C-V,D)	0.0961	0.0983	0.0965	0.0986
ELF(DH-A)	0.0946	0.1438	0.1049	0.1525
CVB index	0.0015	-0.0455	-0.0084	-0.0539

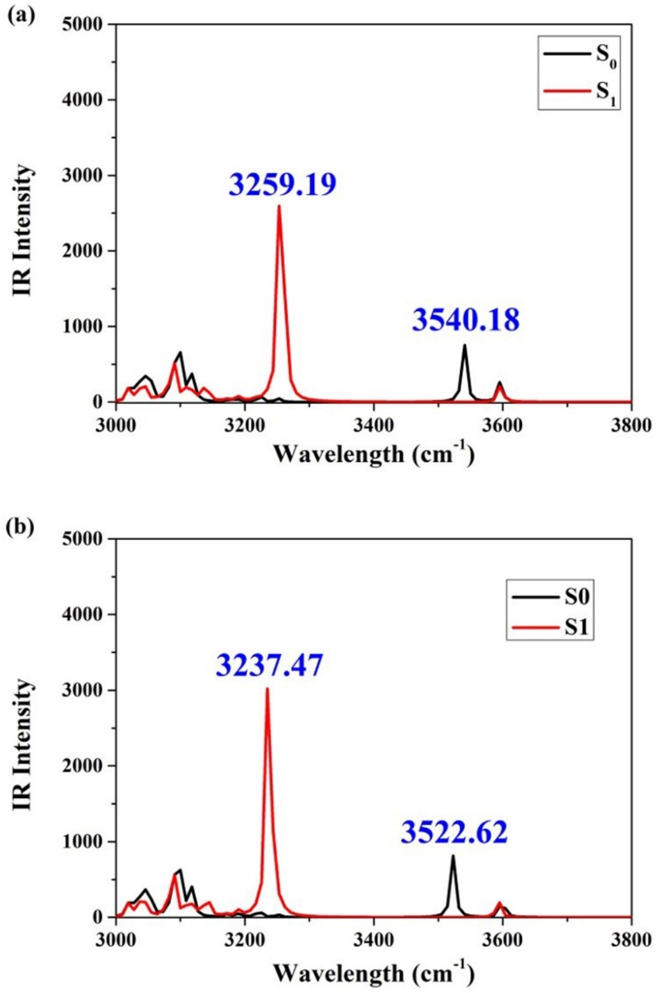

Figure S1. Simulated S_0-state and S_1-state IR associated with the O-H stretching vibrational mode for (a) ENF-S and (b) ENF-Se in dichloromethane solvent, respectively.

The coordinates of all the transition states:

S_1-state TS in cyclohexane

	X	Y	Z
C	1.06394700	0.72558200	-0.16642800
C	-1.16164700	-0.21640900	-0.23690700
C	-1.79195100	1.05516800	-0.11255800
C	-0.95141400	2.16564900	-0.01813400
C	0.51264200	1.98007900	-0.04548900
H	-1.42427200	-2.32010600	-0.43407000

C	-1.92417000	-1.36503300	-0.34003500
C	-3.20927600	1.10746300	-0.09704000
C	-3.96413300	-0.05443100	-0.19946800
C	-3.32230200	-1.29965500	-0.32167400
H	-3.68303000	2.07673200	-0.00308500
H	-3.90976800	-2.19867000	-0.40204600
C	2.45423100	0.35730300	-0.19837400
C	3.48321400	1.32599700	-0.11846500
C	2.85316600	-0.99619700	-0.30456600
C	4.81031900	0.96828900	-0.14699900
H	3.21881600	2.36941400	-0.02745700
C	4.18104300	-1.35921400	-0.33265600
H	2.09723100	-1.76584300	-0.36180000
C	5.21504400	-0.38994600	-0.26471700
H	5.54702000	1.75317200	-0.06984200
H	4.41997800	-2.40903200	-0.40969700
O	0.19775200	-0.36418500	-0.26081500
O	-1.23833500	3.42023600	0.09875600
O	1.08650900	3.14848800	0.05872200
H	-0.02473500	3.71556900	0.11835800
N	6.54038200	-0.74359400	-0.31960500
C	6.94900800	-2.14943200	-0.33971700
C	6.89391300	-2.83514400	1.02853600
H	7.96755600	-2.18110100	-0.72506600
H	6.33063600	-2.69001300	-1.05865000
H	7.16848600	-3.88742800	0.92350200
H	7.58802500	-2.37077600	1.72962100
H	5.89325200	-2.78480000	1.45898600
C	7.60244200	0.26014600	-0.23146700
C	7.93903800	0.68808900	1.19991500
H	7.32198400	1.12772200	-0.82982900
H	8.48673800	-0.16599800	-0.70542300
H	8.69311000	1.47851900	1.18050800
H	7.05713700	1.06854700	1.71678400
H	8.33768800	-0.14647700	1.77752600
N	-5.36798700	0.08780800	-0.16954500
H	-5.68868100	1.03683300	-0.05363800
C	-6.35263100	-0.85839500	-0.28624000
O	-6.14095700	-2.05304900	-0.43352800
C	-7.77011900	-0.30756200	-0.21689700
C	-8.60024900	-1.00714500	0.86601100
H	-8.22558100	-0.48770900	-1.19577100
H	-7.77538600	0.77418600	-0.05400800
C	-10.05303100	-0.53773600	0.87738500

H	-8.55252900	-2.08465100	0.69671000
H	-8.14286400	-0.82304600	1.84333500
H	-10.62486100	-1.04731800	1.65639700
H	-10.12459200	0.53773500	1.06370200
H	-10.54041000	-0.74166800	-0.08017000

S_1-state TS in dichloromethane

	X	Y	Z
C	1.06420600	0.72092100	-0.17769900
C	-1.16306800	-0.22539300	-0.25078400
C	-1.79420600	1.04616700	-0.12504100
C	-0.95307000	2.15826300	-0.03220300
C	0.49938600	1.97729200	-0.05649100
H	-1.42222000	-2.32976500	-0.44719900
C	-1.92272700	-1.37453600	-0.35270200
C	-3.20975500	1.09577000	-0.10732600
C	-3.96602800	-0.06790600	-0.20860000
C	-3.32156000	-1.31164200	-0.33170600
H	-3.69094900	2.06130700	-0.01206000
H	-3.90406800	-2.21398300	-0.41088500
C	2.44881600	0.35967300	-0.20265200
C	3.48031200	1.33108600	-0.11159200
C	2.85363700	-0.99654700	-0.31422700
C	4.80621800	0.97733100	-0.13502600
H	3.21748500	2.37421500	-0.01577400
C	4.17993800	-1.35615800	-0.33743100
H	2.10244800	-1.77048100	-0.38018200
C	5.21511700	-0.38301200	-0.25779700
H	5.54221300	1.76178400	-0.04804000
H	4.42263300	-2.40463000	-0.41943300
O	0.19780700	-0.36996600	-0.27722900
O	-1.24812700	3.42012600	0.08612200
O	1.07602600	3.14759400	0.05034200
H	-0.02596000	3.71199100	0.10793900
N	6.53387200	-0.73296900	-0.30556900
C	6.94832500	-2.14056500	-0.33333300
C	6.88600500	-2.83122500	1.03139200
H	7.96951800	-2.16277800	-0.70964800
H	6.33552700	-2.67820000	-1.05835500
H	7.17463400	-3.87896600	0.91898800
H	7.56870200	-2.36153700	1.73999500
H	5.87940300	-2.79695500	1.45013500
C	7.59862000	0.27239300	-0.21395100

C	7.93947400	0.68221200	1.22096000
H	7.31381000	1.14563200	-0.80061200
H	8.47896000	-0.15234400	-0.69463100
H	8.70080600	1.46569000	1.20241200
H	7.06136000	1.06848600	1.74070400
H	8.33161200	-0.16252000	1.78805500
N	-5.36883700	0.07613500	-0.17411400
H	-5.68944400	1.02525800	-0.04617400
C	-6.35408100	-0.86335500	-0.30387100
O	-6.14074700	-2.05973900	-0.47166200
C	-7.76868700	-0.31529900	-0.21780500
C	-8.57453700	-0.99285600	0.89823300
H	-8.24480800	-0.51641700	-1.18261400
H	-7.76907000	0.76831800	-0.07430600
C	-10.02050600	-0.50437000	0.94339000
H	-8.54926000	-2.07388800	0.74341500
H	-8.08746500	-0.79958400	1.85935100
H	-10.57679400	-0.99785200	1.74413900
H	-10.06996700	0.57415800	1.11864200
H	-10.53499200	-0.71267300	0.00090700

S_1-state TS in acetonitrile

	X	Y	Z
C	1.06335400	0.72213700	-0.18342500
C	-1.16297900	-0.22950200	-0.24913000
C	-1.79682100	1.04232900	-0.13826600
C	-0.95745000	2.15749700	-0.05601900
C	0.49234600	1.97963900	-0.07680400
H	-1.41767000	-2.33684100	-0.41955500
C	-1.91982800	-1.38122400	-0.33728200
C	-3.21206600	1.08914000	-0.12253500
C	-3.96669300	-0.07764500	-0.21065800
C	-3.31913900	-1.32116900	-0.31768700
H	-3.69780600	2.05353700	-0.03934900
H	-3.89889800	-2.22618600	-0.38622200
C	2.44641200	0.36545300	-0.20655500
C	3.47774900	1.33929800	-0.11658700
C	2.85495700	-0.99129900	-0.31599900
C	4.80360700	0.98808500	-0.13633100
H	3.21432900	2.38275300	-0.02665700
C	4.18104100	-1.34841000	-0.33697700
H	2.10629100	-1.76776300	-0.38072400
C	5.21562000	-0.37298300	-0.25491600

H	5.53782400	1.77484800	-0.05652300
H	4.42552700	-2.39674600	-0.41335700
O	0.19846900	-0.37139100	-0.27356200
O	-1.25685400	3.42155600	0.04914000
O	1.06778200	3.15222500	0.01797900
H	-0.03181700	3.71374100	0.06849900
N	6.53295300	-0.71983800	-0.29609800
C	6.95084200	-2.12484300	-0.37968000
C	6.93438500	-2.85455200	0.96562500
H	7.95937500	-2.13139700	-0.78930900
H	6.31586800	-2.64191000	-1.09939300
H	7.21160700	-3.89991300	0.81073100
H	7.64667700	-2.40983200	1.66104100
H	5.94299100	-2.82623700	1.42022600
C	7.59577300	0.28169500	-0.14133800
C	7.85869400	0.68032800	1.31286200
H	7.34680100	1.16059700	-0.73662000
H	8.49836300	-0.14573000	-0.57455100
H	8.63496900	1.44864200	1.34019900
H	6.96017300	1.08388400	1.78200300
H	8.19985000	-0.17446000	1.89742600
N	-5.36913800	0.06410800	-0.17794900
H	-5.69210900	1.01262200	-0.04790800
C	-6.35206500	-0.87608200	-0.31632900
O	-6.13515100	-2.07152500	-0.49289000
C	-7.76702500	-0.33277300	-0.22286000
C	-8.54730000	-0.97921800	0.92972900
H	-8.26024600	-0.56663400	-1.17127900
H	-7.77007000	0.75432000	-0.11157400
C	-9.99227000	-0.48992100	0.99188700
H	-8.52547600	-2.06453700	0.80574500
H	-8.03977400	-0.75728100	1.87391100
H	-10.53112400	-0.96096100	1.81777100
H	-10.03732000	0.59306600	1.13784300
H	-10.52586400	-0.72427000	0.06627600

S_1-state TS of ENF-S in dichloromethane

	X	Y	Z
C	1.17430400	0.22047500	0.09118600
C	-1.46609000	-0.81038800	0.09752300
C	-1.88841300	0.53719900	0.30029500
C	-0.95565600	1.57708400	0.42541800
C	0.50842700	1.41578100	0.30045200

H	-2.10268900	-2.83343200	-0.22542800
C	-2.41789100	-1.80928800	-0.06055700
C	-3.28299200	0.79968400	0.35096900
C	-4.21844600	-0.21347500	0.20991500
C	-3.78321500	-1.53694500	-0.00048000
H	-3.60246100	1.82313700	0.50429700
H	-4.50079000	-2.33205800	-0.11268600
C	2.60473000	0.07393900	-0.08655400
C	3.42341400	1.15526300	-0.50373800
C	3.26608200	-1.16154200	0.13343900
C	4.78022700	1.02074200	-0.66635800
H	2.97006200	2.11551500	-0.69480400
C	4.62300000	-1.31002200	-0.03215400
H	2.70642900	-2.02445500	0.47025800
C	5.44074300	-0.22106700	-0.44030500
H	5.34205000	1.88651300	-0.98177300
H	5.05780600	-2.27622400	0.17179500
O	-1.24339700	2.83696900	0.60956100
O	1.05579400	2.60206300	0.41615500
H	-0.03770700	3.13918700	0.56635900
N	6.78610400	-0.35895800	-0.62000000
C	7.46549200	-1.63284100	-0.35512800
C	7.77864700	-1.86726700	1.12474500
H	8.38963400	-1.62619400	-0.93081900
H	6.85742800	-2.44747600	-0.74897700
H	8.23735600	-2.85142600	1.24539100
H	8.47379600	-1.11694600	1.50230400
H	6.87287300	-1.83392200	1.73192700
C	7.63424900	0.78860600	-0.96712200
C	7.97020700	1.69035700	0.22306100
H	7.14953500	1.36498400	-1.75603300
H	8.55091200	0.38578300	-1.39427700
H	8.57011400	2.53519900	-0.12320200
H	7.06716900	2.08179000	0.69341000
H	8.54324500	1.14883100	0.97611300
N	-5.57753400	0.15114800	0.27730900
H	-5.74061400	1.14296200	0.37883300
C	-6.69942600	-0.63257300	0.24914000
O	-6.67783300	-1.85370300	0.13787600
C	-8.00947400	0.12821100	0.34990800
C	-8.86138700	-0.04667500	-0.91538500
H	-7.84197800	1.19029400	0.54403000
H	-8.54787200	-0.28151300	1.20944600
C	-10.21331500	0.65374900	-0.80080200

H	-8.31087600	0.34775500	-1.77538400
H	-9.00740400	-1.11428800	-1.09591500
H	-10.80414400	0.51772300	-1.70996200
H	-10.79197000	0.25525200	0.03740900
H	-10.09084700	1.72869400	-0.64012200
S	0.23844600	-1.31673000	0.06769400

S_1-state TS of ENF-Se in dichloromethane

	X	Y	Z
C	1.20406700	0.34101100	-0.34132800
C	-1.54000800	-0.75858500	-0.06456800
C	-1.93299100	0.60942300	-0.14134900
C	-1.00715800	1.64842400	-0.34460400
C	0.47046000	1.51306000	-0.40289000
H	-2.19488600	-2.77606700	0.24439800
C	-2.49466300	-1.73641700	0.17551600
C	-3.31338400	0.91106500	0.01422400
C	-4.25696400	-0.08054100	0.23025300
C	-3.84597800	-1.42573500	0.31267300
H	-3.61657200	1.94952000	-0.03582600
H	-4.56994300	-2.20516500	0.48055900
C	2.64694800	0.26058200	-0.29159600
C	3.44241900	1.33499200	0.18704700
C	3.35057600	-0.90561400	-0.68925300
C	4.81285600	1.26601300	0.22922900
H	2.95701800	2.23422000	0.53398700
C	4.72217300	-0.98903500	-0.64793100
H	2.80693300	-1.76317900	-1.06637300
C	5.51538900	0.10508200	-0.20483900
H	5.35226400	2.11391800	0.62297000
H	5.18832000	-1.90555700	-0.97563700
O	-1.32817900	2.91135000	-0.43438600
O	0.97519200	2.71928000	-0.52203000
H	-0.13591800	3.23613600	-0.51669200
N	6.87779800	0.04578700	-0.19839100
C	7.58816900	-1.19714300	-0.52461100
C	7.58712700	-2.22522400	0.60895100
H	8.61228400	-0.92142700	-0.76966800
H	7.15543500	-1.62770300	-1.42895300
H	8.10747600	-3.12723000	0.27854000
H	8.09885500	-1.83698400	1.48994600
H	6.57197400	-2.50228700	0.89601700
C	7.69561700	1.18413000	0.23700800

C	7.91711000	1.24134800	1.74994500
H	7.23457000	2.10606400	-0.11564500
H	8.65442100	1.09978000	-0.27322100
H	8.49489100	2.13484000	1.99792400
H	6.96799700	1.28571300	2.28644000
H	8.47002900	0.36879000	2.09920000
N	-5.60022600	0.32660800	0.35570600
H	-5.74863100	1.31954200	0.24110600
C	-6.72071700	-0.41420700	0.61774100
O	-6.71284700	-1.62888100	0.78702300
C	-8.01108000	0.38419800	0.68031200
C	-9.04730900	-0.12939300	-0.32810000
H	-7.82908000	1.44931100	0.51961400
H	-8.40426200	0.26975700	1.69524200
C	-10.37541600	0.61620600	-0.21953900
H	-8.64442000	-0.02417500	-1.34047600
H	-9.20266000	-1.19740900	-0.15942600
H	-11.09894400	0.23716000	-0.94547800
H	-10.80989900	0.50088500	0.77759200
H	-10.24624700	1.68639000	-0.40496000
Se	0.26380000	-1.35433600	-0.32150000
